# Hematopoietic stem cell specific V-ATPase controls breast cancer progression and metastasis via cytotoxic T cells

**DOI:** 10.18632/oncotarget.26061

**Published:** 2018-09-04

**Authors:** Manoranjan Sahoo, Gajendra K. Katara, Mahmood Y. Bilal, Safaa A. Ibrahim, Arpita Kulshrestha, Sara Fleetwood, Kimiko Suzue, Kenneth D. Beaman

**Affiliations:** ^1^ Department of Microbiology and Immunology, Chicago Medical School, Rosalind Franklin University of Medicine and Science, North Chicago, IL, USA; ^2^ Department of Pathology, Chicago Medical School, Rosalind Franklin University of Medicine and Science, North Chicago, IL, USA

**Keywords:** tumor microenvironment (TME), breast tumor, CD8^+^ T cells, hematopoietic stem cells (HSC), vacuolar ATPase

## Abstract

The interaction of recruited immune effector cells and cancer cells within tumor microenvironment (TME) shapes the fate of cancer progression and metastasis. Many cancers including breast cancer, express a specific vacuolar ATPase (a2V) on their cell surface which acidifies the extracellular milieu helping cancer cell proliferation and metastasis. To understand the role of immune cell-associated-a2V during breast tumor pathogenesis, we knocked-out a2V (KO) from the hematopoietic stem cells (HSC) and generated breast tumors in mice. The a2V-KO mice developed faster growing, larger, and metastatic breast tumors compared to control mice. Further investigation of the TME revealed a significant reduction in the presence of CD4^+^ and CD8^+^ T cells in the a2V-KO tumors. Targeted RNA-Seq of the cells of the TME demonstrated that pro-inflammatory cytokines, death receptors, death receptor ligands, and cytotoxic effectors were significantly down-regulated within the a2V-KO TME. Interestingly, analysis of immune cells in the blood, spleen, and thymus of the non-tumor bearing a2V-KO mice revealed a significant decrease in CD4^+^ and CD8^+^ T cell populations. For the first time, this study demonstrates that inhibition of V-ATPase expression in HSC leads to a decrease in CD4^+^ and CD8^+^ T cell populations and thus promotes breast tumor growth and metastasis.

## INTRODUCTION

The tumor microenvironment (TME) and the interactions between breast cancer cells, immune cells, and their mediators play an important role during tumor progression and metastasis [1]. The TME is infiltrated with various non-immune cells as well as immune cells such as macrophages, dendritic cells, myeloid derived dendritic cells (MDSC), B cells, natural killer (NK) cells and T cells [2–4]. An effective immune response against proliferating tumor cells requires complex interplay among all of the infiltrating immune cells as well as tumor cells. For example, it is well established that both cytokine producing CD4^+^ T helper (T_H_) cells and CD8^+^ cytotoxic T (T_C_) cells are two key players within the TME. The CD4^+^ cells provide necessary factors for priming CD8^+^ cells and for the expansion of tumor-specific CD8^+^ T_C_ cells [5]. CD8^+^ T_C_ cells cause tumor cell apoptosis through different mechanisms such as the direct binding of the Fas ligand to the Fas receptor on tumor cell surface, recognition of specific epitopes on the tumor cell surface, the release of pore forming perforin, and by secreting cell lysing granzymes. In addition, both CD4^+^ T_H_ cells and CD8^+^ T_C_ cells induce cell death indirectly by releasing pro-inflammatory cytokines like IFN-γ, TNF-α and GM-CSF [6, 7]. Tumor cells can also dampen the activation and proliferation of antigen specific CD4^+^ T_H_ cells and CD8^+^ T_C_ cells through binding of programmed death ligand (PD-L1) to the PD-1 receptor on T cells. The binding of PD-1 to PD-L1 results in enhanced apoptosis in the antigen specific CD4^+^ T_H_ cells and CD8^+^ T_C_ cells [8]. Tumor induced MDSCs are also known to suppress the activity, induce tolerance of the various T cells, and cause apoptotic death of CD8^+^ T_C_ cells [9–11]. Furthermore, rapidly proliferating tumor cells acidify the TME, which in turn increases immune cell death and reduces effectiveness of adoptive immunotherapy [12–14].

The vacuolar-ATPase is a multi-subunit H^+^-proton pump that is present on the membrane of intracellular vesicles and maintains intracellular pH in normal cells. A specific isoform of the vacuolar-ATPase, a2V, is also expressed on the surface of cancer cells where it acidifies the extracellular milieu and thus promotes growth, metastasis and chemo-resistance of the cancer cells [15–19]. a2V is encoded by the *ATP6V0a2* gene and its expression is tissue specific [15, 20, 21]. For example, a2V is also expressed in cells of hematopoietic origin such as lymphocytes, monocytes and neutrophils [22–24]. Previous *in vitro* studies have demonstrated that the secreted peptide from cancer-associated a2V, a2V N-terminal domain (a2NTD) modulates IL-1β secretion in THP-1 cells and peripheral blood mononuclear cells [22, 23]. Furthermore, cancer-associated a2NTD modulates the pro-tumorigenic properties of monocytes, macrophages and neutrophils by changing to an alternatively activated phenotype [25–27].

Host-associated a2V also plays an important role during breast cancer progression. The inhibition of host-associated a2V expression in mammary epithelial cells leads to a reduction in glycosylation of the extracellular matrix (ECM), resulting in soft, highly inflammatory and metastatic breast tumors [28]; however, the precise effect of host immune cell-associated a2V inhibition on breast cancer progression is not known. In this study, we generated a conditionally knocked out (KO) mouse model in which expression of a2V was inhibited from the hematopoietic stem cells (HSCs). Following implantation of a syngeneic tumor cell line in the mammary fat pad of mice, the loss of a2V in the HSCs led to enhanced breast tumor growth and metastasis. Investigation of the TME revealed a significant reduction of CD4^+^ and CD8^+^ T cells in the a2V-KO tumors. In addition, targeted RNA-Seq of the TME demonstrated that pro-inflammatory cytokines, death receptors, effector molecules, and pro-apoptotic genes were significantly down regulated, while anti-apoptotic genes remained unchanged. The reduction in recruitment of CD4^+^ and CD8^+^ T cells in the TME is a reflection of T cell populations in the periphery, as seen by analysis of immune cells in the spleen and blood of non-tumor bearing mice. Further investigation of the decrease of T cells in periphery revealed a defect in production of T cells in the bone marrow. Collectively, these results demonstrate, for the first time, that the depletion of HSC-associated a2V leads to a reduction of CD4^+^ and CD8^+^ T cells in the periphery that promotes breast cancer growth and metastasis.

## RESULTS

### Lack of HSC-associated a2V leads to an increase in growth and size of breast tumors

**Figure 1:**
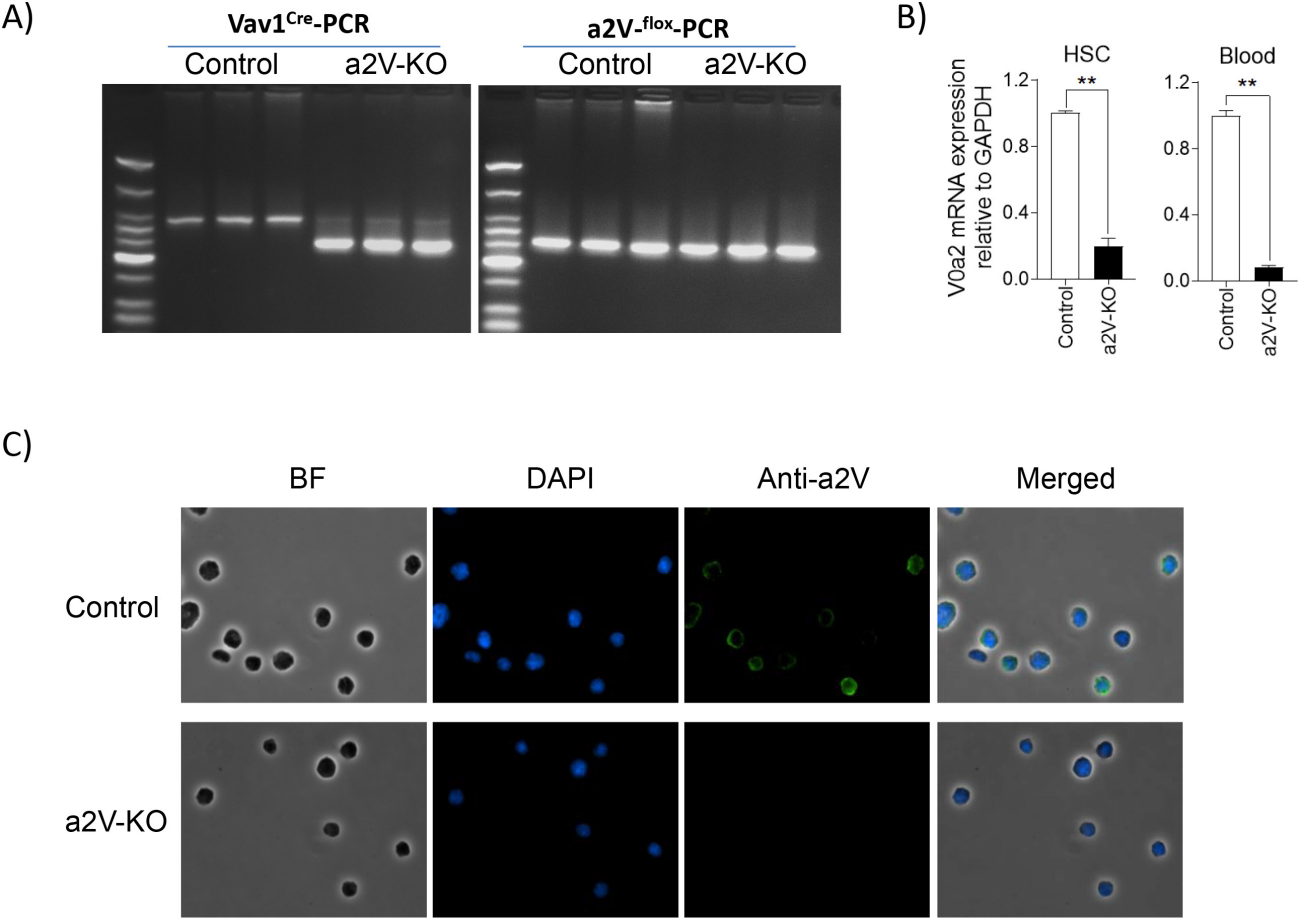
Hematopoietic stem cells lack a2V expression in a2V-KO mice **(A)** Representative genomic DNA PCR gel image for Vav1^Cre^ transgene (left panel) and LoxP sites (right panel) from control (a2V^fl/fl^) and a2V-KO mice (a2V^fl/fl^Vav1^CreTg/0^) (n=3) is shown. **(B)** Relative mRNA level of V0a2 isoform of V-ATPase in HSCs isolated from bone marrow and in white blood cells of mice is shown. Mouse GAPDH is used as an endogenous control for normalization. Data is represented as mean ± SEM (n=6, Mann-Whitney *U* test, ^**^ *p*< 0.01). **(C)** Representative IFA image showing lack of a2V protein (green fluorescence) in HSCs isolated from bone marrow of a2V-KO mice (n=6, 40X magnification).

To understand the role of immune cell-associated a2V in breast cancer pathogenesis, we generated a conditional KO mouse model (Figure 1A) that lacks a2V in all the cells derived from the HSCs. We detected 5 fold and 12 fold reduction in a2V transcript levels in HSCs and in circulating white blood cells, respectively, in a2V-KO (a2V^fl/fl^Vav1^CreTg/0^) mice as compared to control (a2V^fl/fl^) mice (Figure 1B). In contrast, the transcript levels of other isoforms of the ‘a’ subunit, namely V0a1, V0a3, and V0a4, did not show a significant change in HSCs (Supplementary Figure 1A) by qRT-PCR. As demonstrated by IFA, a2V was also visibly absent at protein level in the HSCs collected from bone marrow (Figure 1C) and in *ex vivo* differentiated bone marrow-derived macrophages (Supplementary Figure 1B).

**Figure 2:**
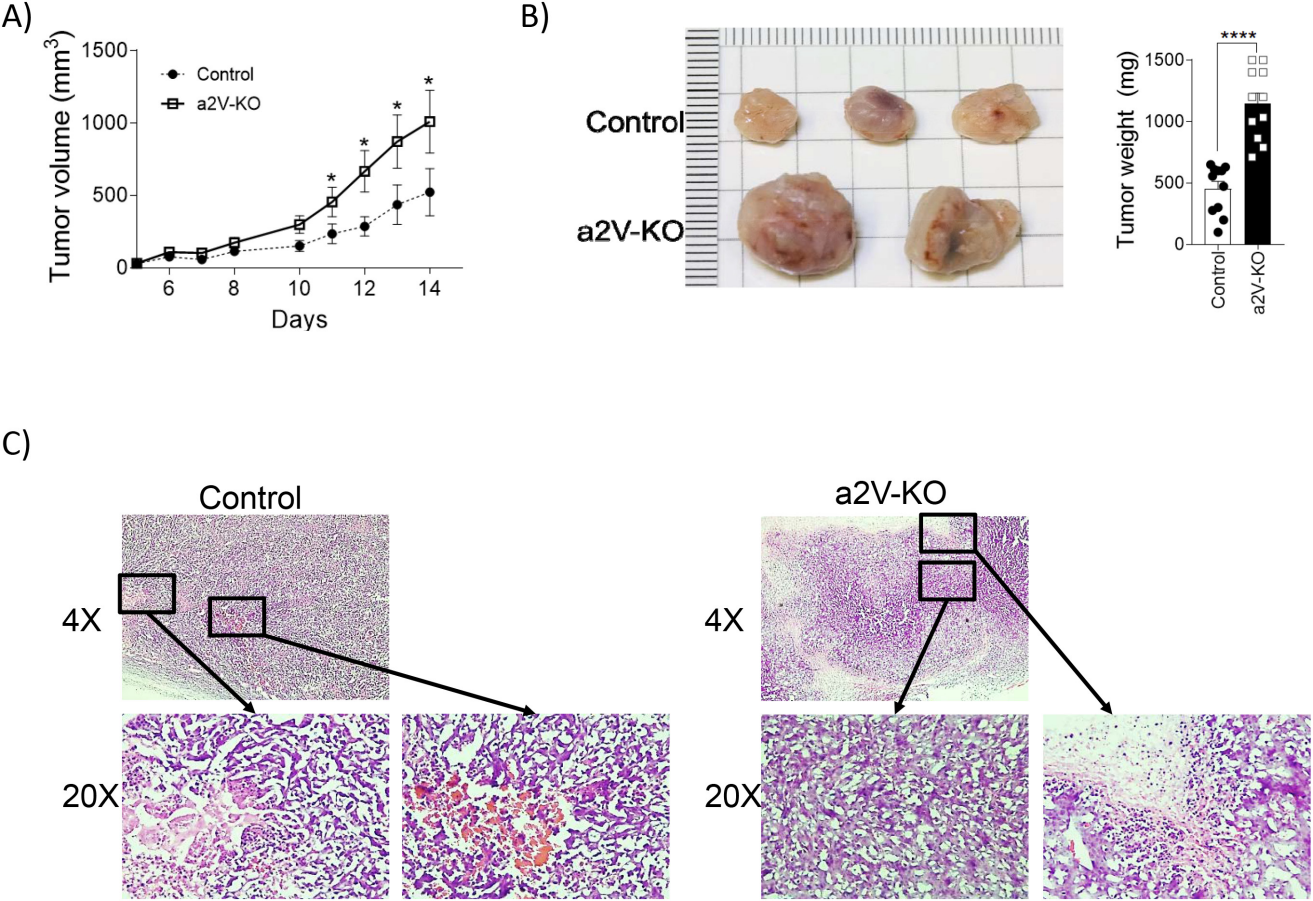
Lack of immune cell-associated a2V leads to an increase in growth and size of breast tumors **(A)** Tumor growth curve: 5x10^5^ E0771 cells were resuspended in 50 μl HBSS and 50 μl Matrigel and were injected into the 4^th^ mammary fat pad of 8 week old female littermate control and a2V-KO mice. Tumor length and width were measured with a digital caliper and tumor volume was determined by the following formula: volume= LxWxWx0.5. Results are shown as mean ± SEM (pooled results from three independent experiments with total n=11, Mann-Whitney *U* test, ^*^p<0.05). **(B)** Left panel shows a representative picture of breast tumors from control and a2V-KO mice collected on day 14 post-implantation. Right panel is graphical representation of pooled tumor weight from 3 independent experiments harvested 14 days post-implantation (n=11, mean ± SEM, Mann-Whitney *U* test, ^****^p<0.0001). **(C)** Representative images of hematoxylin and eosin stained breast tumor sections collected on day 14 post-implantation from control (left panels) and a2V-KO (right panels) mouse. The images in upper panels show overall histopathology at 4X magnification. The images in lower panels are the 20X magnified images from the indicated area. The control tumor sections (left panels) show areas of micro-necrotic and hemorrhagic areas surrounded by tumor and infiltrating cells. In the a2V-KO tumors (right panels), the tumor cell zones are surrounded by large necrotic pink areas with pyknotic and karyolytic cells.

To understand how the deletion of a2V in HSC affects breast cancer progression, we implanted a syngeneic tumor cell line, E0771 into the 4^th^ mammary fat pad of control and a2V-KO mice, both of which share the same C57BL/6 background. Tumor growth analysis showed that tumors in a2V-KO mice grew rapidly at a faster rate as evident by the higher mean slope of growth curve of 81.9mm^3^/Day ± SEM 8.779mm^3^/Day in a2V-KO mice while mean slope of 31.6mm^3^/Day ± SEM 5.979mm^3^/Day in the control mice (Figure 2A). The isolated tumors from both control and a2V-KO mice appeared solid upon inspection and were phenotypically similar except for size and mass (Figure 2B). The tumors of a2V-KO mice grew 2.5 times larger in mass compared to the control mice on the 14^th^ day post-implantation (mean 1145 mg ± SEM 86.5 mg vs mean 456.6 mg ± SEM 59.6 mg; *p*<0.0001; Figure 2B). To further investigate the tumors, histopathological evaluation of H&E stained tumor sections was performed. The control tumors showed confluent layers of viable tumor (large cuboid) cells with microscopic foci of necrotic (pink area devoid of nuclei) areas and also micro-hemorrhagic (red) areas. The edges of the necrotic areas were heavily infiltrated with higher number of mononuclear cells and few polymorphonuclear cells (Figure 2C). On the other-hand, the tumors from a2V-KO mice showed large areas of viable tumor foci surrounded by necrotic zones. The necrotic zones were larger in area and were filled with pyknotic cells and cells showing karyolysis. Many tumor sections also displayed zones of hemorrhage (not shown). Furthermore, the a2V-KO tumors also displayed a large infiltration of mononuclear cells around the necrotic zones (Figure 2C). The faster growing and larger tumors in a2V-KO mice suggest that the infiltrating immune cells are not efficient in restricting tumor cell proliferation.

### a2V deletion in HSCs cause altered recruitment of immune cell populations into the TME

**Figure 3:**
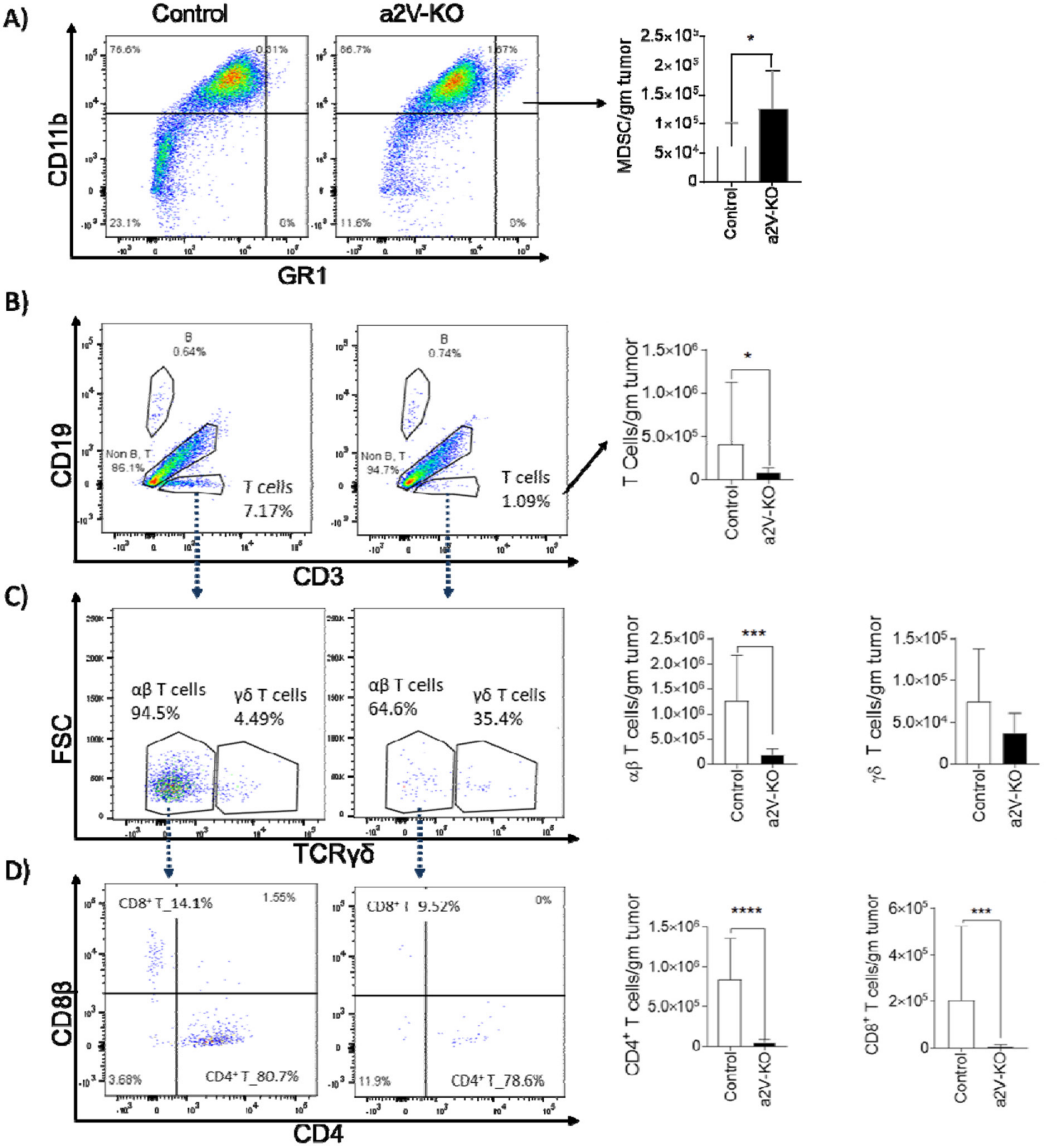
a2V deletion in HSCs cause altered recruitment of immune cell populations into the TME After 14 days of implantation, tumors from control and a2V-KO mice were harvested, mechanically disrupted, and digested with Collagenase IV and DNAse I to obtain a single cell suspension. The single cells were enriched for CD45 expression by MACS and subjected to flow-cytometry and counted manually with trypan blue. Representative histograms are shown in the left side. Bar graphs show the number of single, live, CD45^+^ cells per gram of tumor in the TME. **(A)** MDSC **(B)** T cells **(C)** αβ T cells and γδ T cells **(D)** CD4^+^ T_H_ cells and CD8^+^ T_C_ cells. Pooled results from three independent experiments with control n=14 and a2V-KO n=11, mean ± SEM, Mann-Whitney *U* test, ^*^p<0.05, ^**^p<0.01, ^***^p<0.001, and ^****^p<0.0001.

To understand the TME of a2V-KO tumors, we analyzed the number of infiltrated CD45^+^ cells by flow-cytometry. The majority of the infiltrated CD45^+^ cells were of myeloid origin (∼75% CD11b^+^ cells in control mice and ∼85% CD11b^+^ cells in a2V-KO mice, data not shown). The CD11b^+^ myeloid cells were further identified as CD11b^+^F4/80^+^ tumor associated macrophages (TAM), CD11b^+^GR1^+^ MDSC, CD11b^+^Ly6G^+^ tumor associated neutrophils (TAN). There was no significant difference (*p*=0.7) in the number of TAMs between the TME of control and a2V-KO mice (Supplementary Figure 2A). However, there were twice more MDSC (mean 61,380 ± SEM 10,598 vs mean 126,606 ± SEM 20,735; *p*=0.022, Figure 3A) were present in the a2V-KO TME. Although, the number of TANs displayed a similar trend with mean 66,800 ± SD 14,485 in control TME vs mean 82,250 ± SD 32,375 in a2V-KO TME; *p*=0.3810, there was no significant difference between the groups (Supplementary Figure 2D). In contrast, we found a significant difference in the infiltrated lymphoid cell populations. There was a 3.26 fold increase in recruitment of lymphoid cells in control tumors compared to the a2V-KO tumors. Further analysis demonstrate that there was no significant difference in the CD19^+^CD3^-^ B cell populations (*p*=0.36, Supplementary Figure 2B) and CD19^-^CD3^-^NKp46^+^ NK cell populations (*p*=0.064, Supplementary Figure 2C). On the other hand, the number of CD3^+^CD19^-^ T cell populations were significantly reduced in a2V-KO TME (mean 407,749 ± SEM 173,278 vs mean 75,288 ± SEM 17,374; *p*<0.0389, Figure 3B) compared to the TME of control mice. The reduced number of CD3^+^CD19^-^ total T cells was due to the significantly reduced number of the CD3^+^CD19^-^γδTCR^-^ αβ T cells (*p*<0.0001, Figure 3C). However, this reduction of CD3^+^CD19^-^ total T cells was not due to difference in CD3^+^CD19^-^γδTCR^+^ γδ T cell population (*p*=0.1698, Figure 3C). Further analysis of the αβ T cells revealed significant reduction in both CD4^+^ T_H_ (*p*<0.0001) and CD8^+^ T_C_ cell (*p*<0.0002) populations in the TME of a2V-KO mice. The mean number per gram of CD4^+^ T_H_ and CD8^+^ T_C_ cells was found to be 841,486 ± SEM 127,272 and 202,010 ± SEM 77,719%, respectively, in control mice. In contrast, mean number per gram of CD4^+^ T_H_ and CD8^+^ T_C_ cells in a2V-KO mice were 49,503 ± SEM 10,041 and 5,579 ± SEM 2,532, respectively (Figure 3D). These results suggest that the higher occurrence of MDSC along with reduced number CD8^+^ T_C_ cells and the cytokine producing CD4^+^ T_H_ cells within the TME of a2V-KO mice can be responsible for the larger and faster growing tumor in those mice.

### Tumors of a2V-KO mice develop a pro-tumorigenic environment

**Figure 4:**
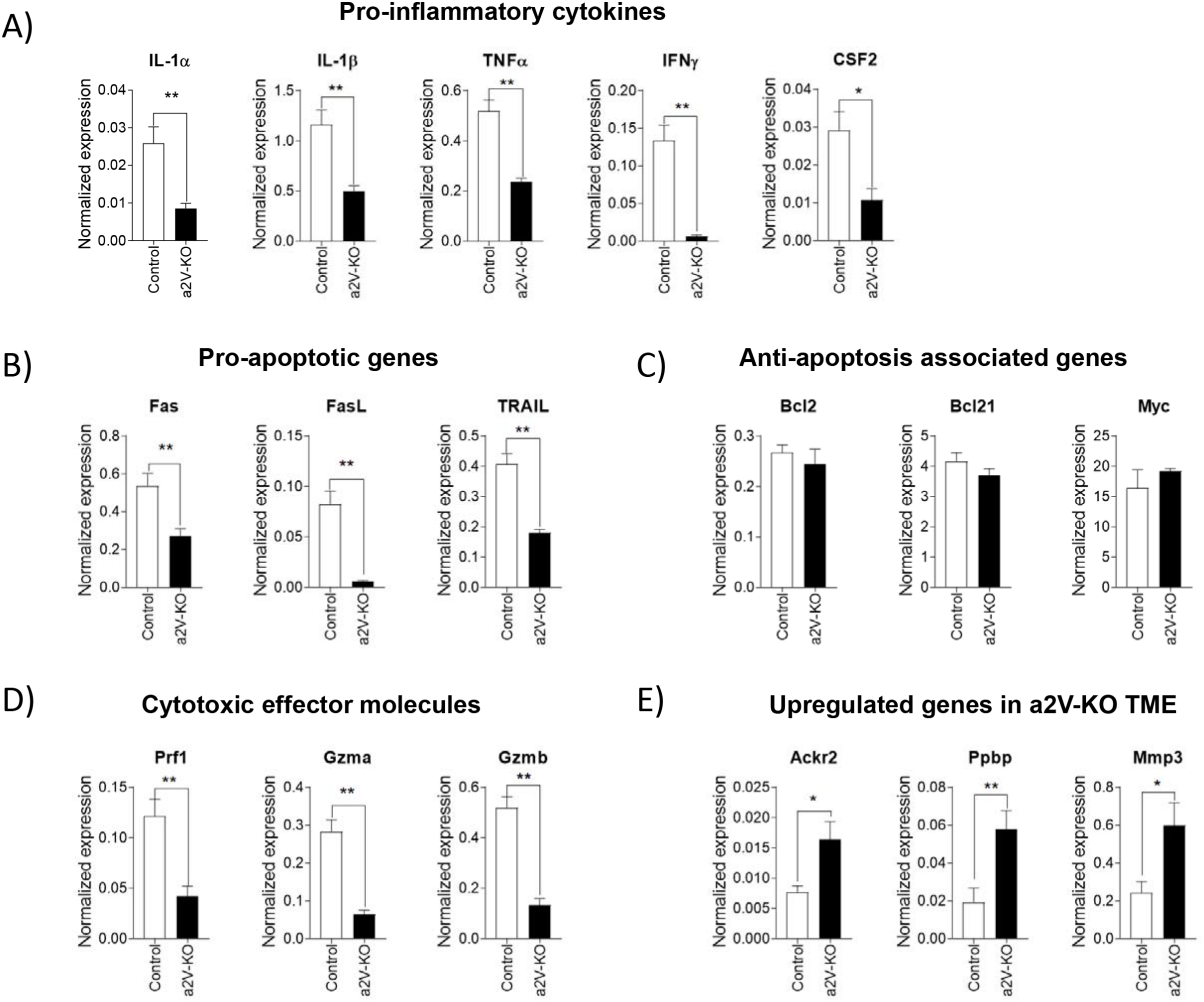
Tumors of a2V-KO mice develop a pro-tumorigenic environment Equal amounts of tumor RNA was used for targeted RNA-Seq (Miseq) and data were analyzed online using Qiagen’s data analysis center. Eight housekeeping genes were used to normalize gene expression. Bar graphs show transcript levels of **(A)** Pro-inflammatory cytokines **(B)** Pro-apoptotic genes **(C)** Anti-apoptosis associated genes **(D)** Cytotoxic effector molecules **(E)** the 3 upregulated genes in a2V-KO TME. Pooled results from two independent experiments with n=6, mean ± SEM, Mann-Whitney *U* test, ^*^p<0.05, and ^**^p<0.01).

To better understand the cytokines and chemokines that contribute to the dynamics of the TME, we performed targeted RNA-Seq via Next Generation Sequencing (NGS). Here we analyzed 485 genes out of which 3 genes were significantly upregulated while 144 genes were significantly downregulated (Supplementary Figure 3A). This analysis uncovered that the transcripts of pro-inflammatory cytokines for IL-1α (3.03 fold, *p*=0.0022), IL-1β (2.34 fold, *p*=0.0043), TNF-α (2.1 fold, *p*=0.0022), IFN-γ (21.3 fold, *p*=0.0022), GM-CSF (2.67 fold, *p*=0.0152) were significantly decreased in the a2V-KO TME (Figure 4A). The results indicate that the TME of control mice is pro-inflammatory and creates an anti-tumorigenic niche. In contrast, the TME of a2V-KO mice is not as pro-inflammatory in nature, and thus creates a pro-tumorigenic environment which is conducive for tumor growth (Figure 4A). The pro-inflammatory cytokines like TNF-α, IFN-γ and GM-CSF are known to induce cell death indirectly by increasing expression of death receptors and death ligands on cells [6, 7]. Our results demonstrate that the transcript levels of pro-apoptotic death receptor, Fas (1.95 fold, *p*=0.0087), death receptor ligands like Fas ligand (FasL; 14.2 fold, *p*=0.0022) and TNF-related apoptosis-inducing ligand (TRAIL; 2.3 fold, *p*=0.0022) are significantly decreased in the a2V-KO tumors (Figure 4B). This data suggests that, the tumor cells of a2V-KO mice are not undergoing apoptosis, and therefore lead to a larger tumor. Furthermore, we also found that there is no difference in the anti-apoptotic transcript levels of Bcl2 (*p*=0.1385), Bcl21 (*p*=0.1320), and Myc (*p*=0.0931). This result also implies that the larger size of the a2V-KO tumors is not due to any protective effect of anti-apoptotic genes; rather it is due to a lack of pro-apoptotic factors in the TME (Figure 4C). This data is also supported by our findings that there is less recruitment of CD8^+^ T_C_ cells (Figure 3D and Supplementary Figure 3B) in to the TME of a2V-KO mice. When compared to the control TME, there is a significant decrease in transcript numbers of cytotoxic effector molecules like perforin (2.89 fold, *p*=0.0087), granzyme-a (4.38 fold, *p*=0.0022), and granzyme-b (3.87 fold, *p*=0.0022) in the TME of a2V-KO mice (Figure 4D). The 3 genes whose transcript levels were significantly increased in the a2V-KO TME are: *Ackr2* (Atypical chemokine receptor 2, D6; 2.1 fold higher, *p*=0.026), *Ppbp* (Pro-platelet basic protein, CXCL7; 3.0 fold higher, *p*=0.0087), and *Mmp3* (Matrix metalloproteinase-3; 2.5 fold higher, *p*=0.0152) (Figure 4E). D6 is a chemokine decoy receptor which sequester pro-inflammatory chemokines [29] while both CXCL7 and MMP3 can degrade the extracellular matrix and promote tumor growth and metastasis [30, 31]. Taken together, the lack of cytotoxic cells, cytotoxic effector molecules, and pro-apoptotic molecules within a pro-tumorigenic environment contributed to the large tumor in the a2V-KO mice.

### Lungs of a2V-KO mice show increased metastasis

**Figure 5:**
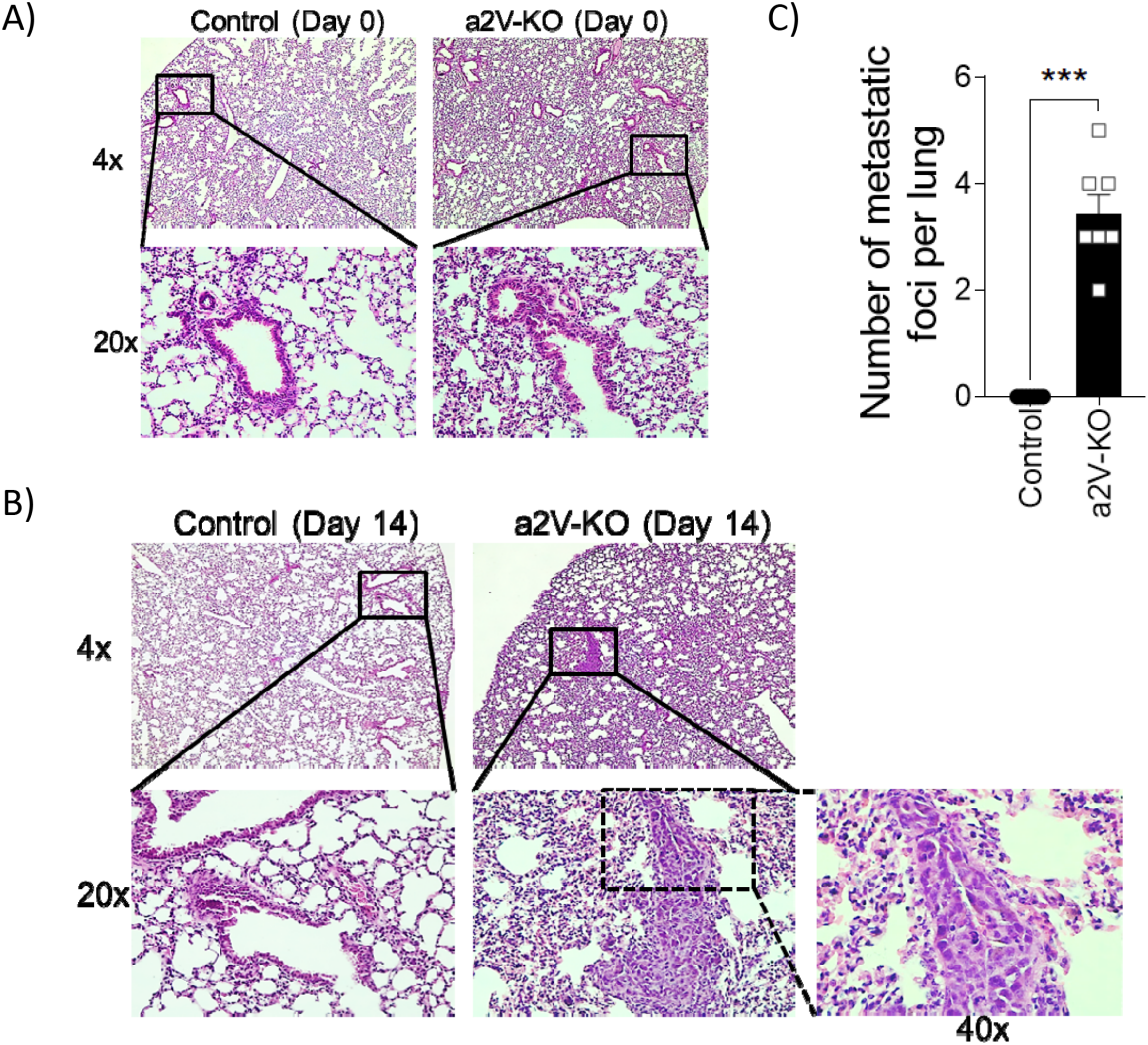
Lungs of a2V-KO mice show increased metastasis Representative histopathology images of hematoxylin and eosin stained mouse lung sections collected on day 0 **(A)** or day14 **(B)** post-implantation from control (left panels) and a2V-KO mice (right panels). The upper panels are at 4X magnification, while the lower panels are the 20X magnified images from the indicated solid lined area or 40X (dotted line from 20X image). **(C)** Quantification of metastatic foci per H&E stained lung sections as observed at 20X magnification on day 14 post-implantation. Day-14 post-implantation lungs are from two independent experiments with n=7, mean ± SEM, Mann-Whitney *U* test, ^***^p<0.001, while day 0 control lungs are from n=5 mice.

To understand if the loss of a2V in the hematopoietic cells impacts the ability of tumor cells to metastasize, we searched for metastatic cancer cells forming a distinct focus in the lungs of a2V-KO and control mice. Time point analysis of cancer metastasis show that the lungs from non-tumor bearing control or a2V-KO mice did not have any metastasis (Figure 5A). However, on day 14 of post-tumor-implantation, breast tumor cells metastasized to the lungs of a2V-KO mice but not into the lungs of control mice. The implanted E0771 tumor cells form significantly higher (*p*=0.006) numbers of metastatic foci of cancer cells that are visibly different from the surrounding lung epithelial cells (Figure 5B and 5C). These results showed that the breast tumors of a2V-KO mice metastasize rapidly compared to control mice, suggesting a less restrictive TME that permits breast cancer cells to metastasize easily.

### Deletion of a2V in hematopoietic cells results in reduced population of αβ T cells

**Figure 6:**
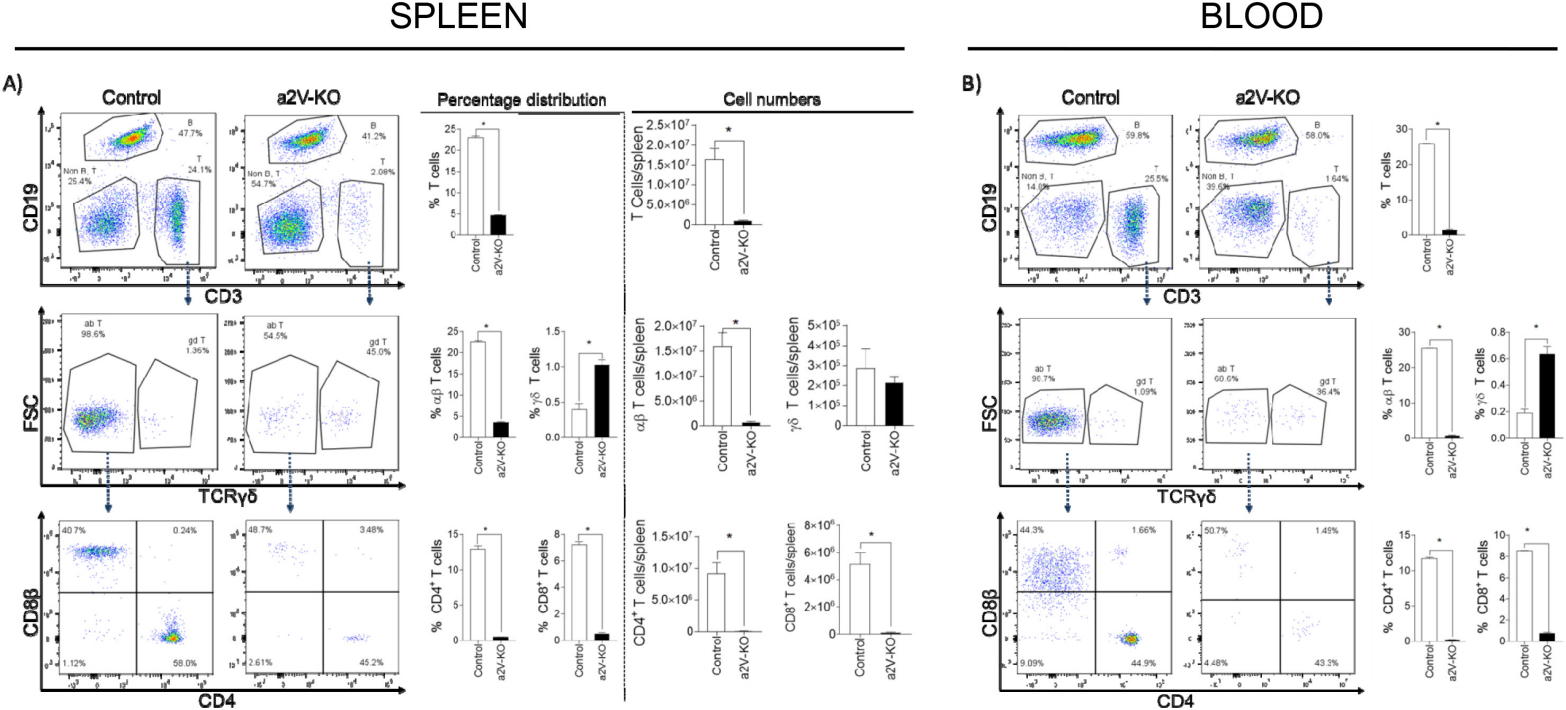

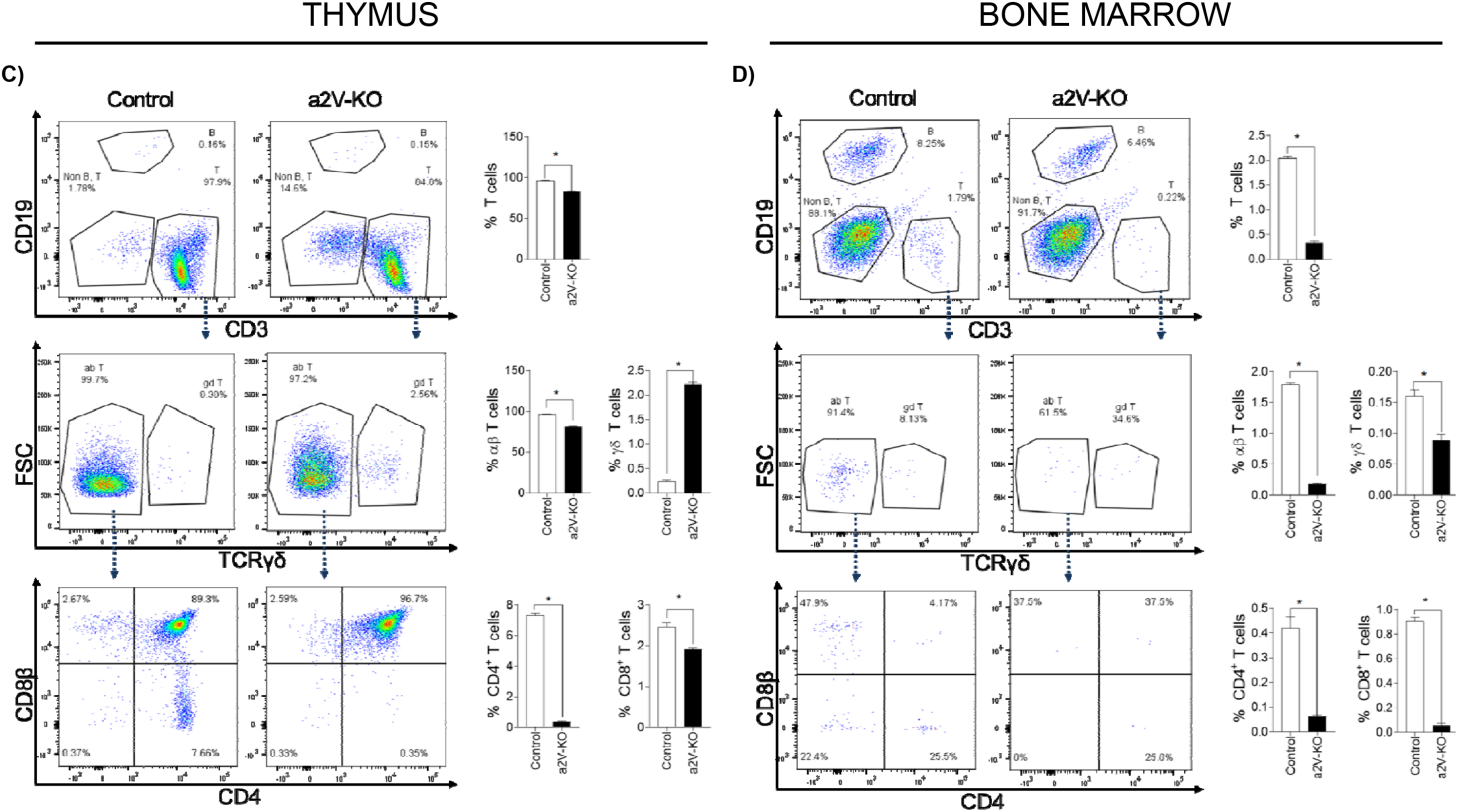
Deletion of a2V in hematopoietic cells results in reduced population of αβ T cells Spleen, thymus and flushed bone marrow were collected from control and a2V-KO non-tumor bearing mice and mechanically disrupted to obtain a single cell suspension. Blood was collected with EDTA to prevent clotting. After lysing red blood cells, the cells were subjected to flow-cytometry after live-dead staining. Representative histogram panels for all the samples are shown in the left. Bar graphs show the percentage **(A;** left panels**)** and number **(A;** right panels**)** of live single cells in the spleen. Furthermore, panels **(B**, **C**, and **D)** show the percentage of live single cells in blood, thymus, and bone marrow, respectively. The upper rows represent T cells, middle rows for αβ T cells and γδ T cells, and bottom rows represent CD4+ T_H_ cells and CD8+ T_C_ cells. Representative experiment with n=4, mean ± SD, Mann-Whitney *U* test, *p<0.05.

HSCs are the progenitors of most immune cell populations. To understand if the change in immune cell populations in TME is due any intrinsic defect in immune cell development, we analyzed the splenic and circulating immune cell populations of non-tumor bearing mice by flow-cytometry. The macrophages and MDSC cells are present in a very low percentage in both spleen and blood (data not shown). As expected, the lymphoid cells constituted the majority cell population within the spleen and blood. The population of lymphoid cells in the a2V-KO spleen was severely diminished. The percentage of total T cells was reduced 4.8 fold and 16.6 fold in spleen and blood of the a2V-KO mice, respectively (Figures 6A and 6B, upper rows). As observed within the TME, only the αβ T cells, not the γδ T cells were diminished in the spleen and blood of a2V-KO mice (Figures 6A and 6B, middle rows). In the spleen, the αβ T cell percentage was reduced 6.23 fold of a2V-KO mice (mean 22.57% ± SD 0.31% in control mice vs mean 3.62% ± SD 0.12% in a2V-KO mice) while the αβ T cell number was reduced 21 fold (mean 16x10^6^ ± SD 2.6x10^6^ in control mice vs mean 0.76x10^6^ ± SD 0.13x10^6^ in a2V-KO mice). Similar reduction of αβ T cell percentage was observed in blood of a2V-KO mice (29.23 fold, mean 25.6% ± SD 0.14% in control mice vs mean 0.88% ± SD 0.27% in a2V-KO mice). Further analysis of the αβ T cells revealed that the percentage of both CD4^+^ T_H_ and CD8^+^ T_C_ cells were reduced 25.71 fold and 13.73 fold in spleen, 58.3 fold and 11.16 fold in blood, respectively (Figure 6A and 6B, bottom rows). The percentage of splenic CD4^+^ T_H_ cells was determined to be (mean ± SD) 12.94% ± 0.35% in control mice, while the percentage (mean ± SD) in a2V-KO mice was found to be 0.50% ± 0.02%. The number of splenic CD4^+^ T_H_ cells was determined to be (mean ± SD) 9.2x10^6^ ± 1.7x10^6^ in control mice, while the number of splenic CD4^+^ T_H_ cells (mean ± SD) in a2V-KO mice was determined to be 1.06x10^5^ ± 0.2x10^5^. The percentage of blood CD4^+^ T_H_ cells was found to be (mean ± SD) 11.71% ± 0.32% in control mice, while the percentage (mean ± SD) in a2V-KO mice was found to be 0.20% ± 0.07%. The mean ± SD percentage of splenic CD8^+^ T_C_ cells was found to be 7.24% ± 0.21% in control mice, compared to 0.53% ± 0.77% in a2V-KO mice, and the mean number ± SD of splenic CD8^+^ T_C_ cells was found to be 5.1x10^6^ ± 0.8x10^6^ in control mice, compared to 1.1x10^5^ ± 0.35x10^5^ in a2V-KO mice, while the mean ± SD percentage of CD8^+^ T_C_ cells in blood was measured to be 8.53% ± 0.0.08% in control mice, compared to 0.76% ± 0.18% in a2V-KO mice. Similar reduction is observed in thymus and bone marrow (Figure 6C and 6D). The reason for such reduction is currently unknown. We are currently working to understand the reasons behind such reduction of the αβ T cells, but it is beyond the scope of this manuscript.

### Depletion of CD8^+^ cytotoxic T cells in periphery leads to increased tumor growth

**Figure 7:**
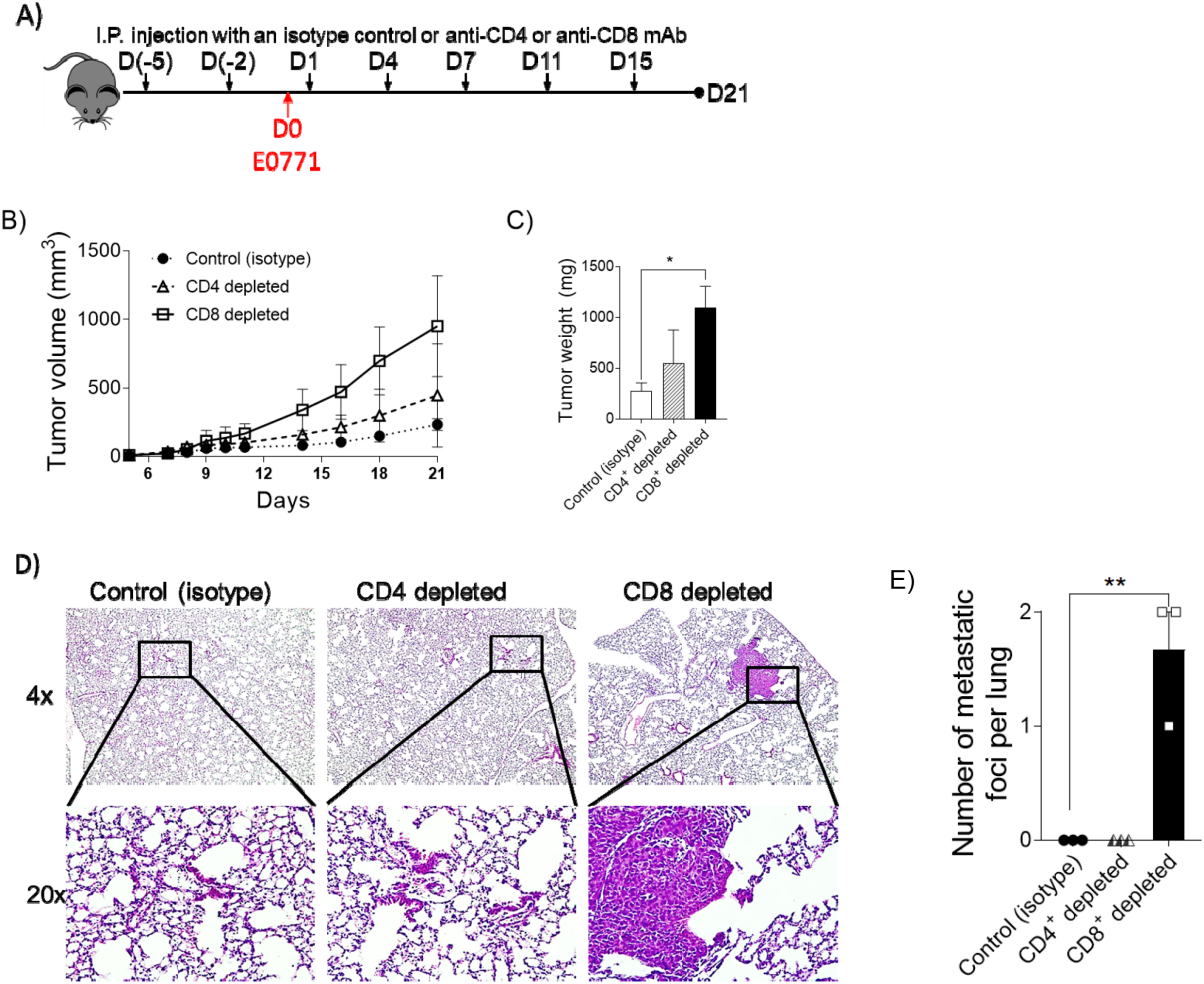
Depletion of CD8^+^ cytotoxic T cells in periphery leads to increased tumor growth **(A)** Diagram showing the scheme for administration of anti-CD4 (n=3) or anti-CD8 (n=3) or an isotype control (n=3) and tumor implantation. **(B)** Tumor growth was measured by digital caliper and is represented as tumor volume (LxWxWx0.5). **(C)** Tumors were harvested on day 21 post-implantation and weighed. Data is presented as bar graph, unpaired *t*-test, ^*^p<0.05. **(D)** Representative images of hematoxylin and eosin staining of lung sections collected on day 21 post-tumor-implantation from control (left panels), CD4 depleted (middle panels) and CD8 depleted (right panels) mice. The upper panels are at 4X magnification, while the lower panels are the 20X magnified images from the indicated solid lined area. **(E)** Quantification of metastatic foci per H&E stained lung sections as observed at 20X magnification on day 21 post-implantation. Day-21 post-implantation lungs are from one experiment with n=3, mean ± SD, unpaired t-test, ^**^p<0.01.

We demonstrated that the diminished occurrence of CD4^+^ T_H_ cells and CD8^+^ T_C_ cells in the a2V-KO tumor (Figure 3D) as well as in the periphery (Figures 6A and 6B, bottom rows) creates a pro-tumorigenic environment that is conducive for tumor growth and metastasis. To understand the relative contribution of CD4^+^ T_H_ cells and CD8^+^ T_C_ cells in controlling tumor growth and metastasis, we depleted either CD4^+^ T_H_ cells or CD8^+^ T_C_ cells from the control mice. The specific depletion was achieved by administration of specific monoclonal antibodies against CD4 or CD8 antigens or with an isotype matched antibody that was used as a control, before tumor implantation. The depleted status was maintained by recurring administration of the above mentioned antibodies as shown in the schematic (Figures 7A and Supplementary Figure 4). We observed that depletion of CD8^+^ T_C_ cells resulted in the most rapidly growing tumors with a mean slope of 59.89 mm^3^/Day ± 5.079 mm^3^/Day (R^2^=0.9456), while depletion of CD4^+^ T_H_ cells yielded moderate results with a mean slope of 25.22 mm^3^/Day ± 2.319 mm^3^/Day (R^2^=0.9367); Control mouse tumor growth was the slowest with a mean slope of 12.5 mm^3^/Day ± 1.321 mm^3^/Day (R^2^=0.918; Figure 7B). In addition, depletion of CD8^+^ T_C_ cells resulted in the largest tumors (mean 1095 mg ± SD 365 mg) and grew 3.94 times bigger when compared to the tumors of control mice. In contrast, depletion of CD4^+^ cells resulted in tumors of mean 552 mg ± SD 560.4 mg and were 1.99 times bigger compared to the control mice (Figure 7C). To appreciate the effect of depletion on metastasis, the lungs were collected and histological analysis was performed as described earlier. Lungs of mice with depleted CD8^+^ T_C_ cells, show metastatic foci while lungs of control mice or mice depleted of CD4^+^ T_H_ cells did not show any distinct foci of metastasis (Figure 7D). These findings show that lack of CD8^+^ T_C_ cells in the periphery result in faster growing, larger tumors that metastasize to lungs.

## DISCUSSION

V-ATPase is expressed on the surface of breast cancer cells and promotes cancer cell growth, invasion and metastasis [19, 32, 33]. Few studies have looked into the role of cancer-associated V-ATPAse during breast cancer progression and all of them agree that deletion of V-ATPase in cancer cells results in a delay in cancer proliferation and invasion [19, 32, 33]. However the effect of immune cell-associated a2V on cancer cell proliferation and metastasis is not known. Deciphering the role of immune cell-associated V-ATPase during breast cancer progression could provide us with a better understanding of other mechanisms that contribute to cancer pathogenesis and metastasis. The present study examines the role of host immune cell-associated V-ATPase during breast tumor pathogenesis by using a mouse model that lacks a2V in the HSCs. Here for the first time we demonstrate that the deletion of a2V in the HSC leads to a significant reduction of CD4^+^ and CD8^+^ T cells in the periphery, which in turn, promotes breast cancer growth and metastasis.

Majority of the immune cell populations originate from HSCs, while a few cell types are originated in fetal liver [34]. To understand the role of host immune cell-associated a2V on breast tumor pathogenesis, we developed a2V-KO mice model that lacks a2V in HSCs and used E0771 to generate tumors as the E0771 tumors mimic the human disease and metastasize [35]. Deletion of HSC-associated a2V has significant effect on tumor growth and metastasis (Figure 2A and 2B). Although, tumors from both control and a2V-KO mice are phenotypically similar except for size and mass, histologically there are major differences between them. The tumors from control mice have multiple micro-necrotic centers and micro-hemorrhagic areas surrounded by infiltrating leukocytes. Similar findings also have been observed in E0771 tumors generated in other mouse models [35] and in human medullary breast cancer [36]. The necrotic zones are of larger size and form a ring shape around the cancer cells in the a2V-KO mice. It is plausible that as the tumor grows larger in mass; the cancer cells migrate outwards, thus creating a necrotic center. Although both control and a2V-KO mice show infiltration of immune cells in the TME, the infiltrating cells of the control mice must be very important in restricting tumor cell proliferation, thus leading to a smaller size tumor. In contrast, the infiltrating cells of the a2V-KO mice may not be very efficient in controlling tumor cell proliferation, resulting in a larger and faster growing tumor.

The complex interaction between the CD4^+^ T_H_ cells and CD8^+^ T_C_ cells and other cell types in the TME impacts the outcome of cancer progression and metastasis. We have found that the presence of fewer CD4^+^ T_H_ cells and CD8 T_C_ cells in the TME correlates with larger tumor sizes in a2V-KO mice (Figure 3D). Our findings are supported by Hannen et al, who reported that the presence of the CD4^+^ T_H_ cells and CD8^+^ T_C_ cells within the TME is critical to control tumor growth [37]. In addition, MDSCs suppress the activity of T cells and induce apoptosis [38]. Our results show that the lower number of CD4^+^ T_H_ cells and CD8^+^ T_C_ cells as well as higher numbers of MDSCs in a pro-tumorigenic environment may lead to the increased growth of tumors in the a2V-KO mice.

To better understand the immune-regulatory factors that contribute to the dynamics of the TME, we performed targeted RNA-Seq. We analyzed 485 genes out of which 144 genes were significantly downregulated while 3 genes were significantly upregulated in a2V-KO TME (Supplementary Figure 2A). The findings from targeted RNA-Seq experiments further support our hypothesis that the TME of a2V-KO mice is pro-tumorigenic (*i.e.* less inflammatory) than the control TME. The a2V-KO mice TME has significantly lower levels of pro-inflammatory cytokine transcripts (Figure 4A), which correlates very well with lower occurrence of CD4^+^ T_H_ cells in the a2V-KO TME. The lower percentage of CD4^+^ T_H_ cells and CD8^+^ T_C_ cells in a2V-KO TME is also reflected by a significant decrease in T cell- and immune-check-point- related transcripts in a2V-KO TME (Supplementary Figures 3B & 3C). However, we did not find any significant difference in the transcript level for CD4 between a2V-KO and control TMEs. CD4 is also expressed on macrophages and there is no significant difference between TAM populations of a2V-KO and control TME (Supplementary Figure 2A). Pro-inflammatory cytokines like TNF-α, IFN-γ and GM-CSF upregulate death receptors on cancer cells and induce apoptosis [6, 7]. Our results demonstrate that in comparison to the control TME, there is also a significantly lower level of transcripts of death receptors and cytotoxic effector in the a2V-KO TME (Figures 4B and 4D). These results bolster the idea that the a2V-KO TME is pro-tumorigenic and conducive for tumor growth, as there are significantly less pro-inflammatory cytokines, cytotoxic effector molecules and T_C_ cells compared to the control TME. As a group, these factors contribute to a faster growing and larger breast tumor in the a2V-KO mice.

Transcript levels of *Ackr2*, *Mmp3*, and *Ppbp* are significantly increased in the a2V-KO TME (Figure 4E). Atypical chemokine receptor 2 or D6 is a decoy receptor or scavenger receptor that sequesters various chemokines by high affinity binding [29, 39, 40]. Higher transcript levels of *Ackr2* indicate lower transcript levels of chemokines present in the a2V-KO TME. In fact, this was found to be true (Supplementary Figure 3D). The pro-platelet basic protein or CXCL7 is a chemokine and acts as heparanase [30]. MMP3 is expressed in higher levels breast cancer [41] and involved in mammary carcinogenesis [31]. It has been reported that higher levels of MMP3 is viewed as a marker of unfavorable prognosis in invasive breast cancer [42]. Furthermore, malignant breast cancer cells express higher levels of CXCL7 and it helps in invasion and metastasis [43]. CXCL7 is also important for self-renewal of breast cancer stem cells [44]. The higher expression levels of D6 sequesters available pro-inflammatory chemokines from the a2V-KO TME making it less inflammatory, while higher expression of MMP3 and CXCL7 help tumor cells to degrade extracellular matrix and metastasize. Collectively, these results support our previous findings that the a2V-KO TME is not inflammatory, and thus is conducive for cancer cell proliferation and metastasis.

The presence of both the CD4^+^ T_H_ cells and CD8^+^ T_C_ cells in the periphery is very important to control tumor progression and metastasis. However, the extent of protection offered by the two major subtypes of T cells, namely the CD4^+^ T_H_ cells and CD8^+^ T_C_ cells varies. It has been reported that at different time points, both the CD4^+^ T_H_ cells and CD8^+^ T_C_ cells could have similar or opposing roles during breast cancer progression. At later time points, the composition of CD4^+^ T_H_ cells differentiates from a T_H_1 phenotype to T_H_17 phenotype, resulting in negative prognostic effects, while the CD8^+^ T_C_ cells are the key effector cells that control tumor progression [45, 46]. As shown in the T cell depletion experiment (Figure 7), the lack of CD8^+^ T_C_ cells in the periphery had the most effect on tumor growth and metastasis. In contrast, the lack the CD4^+^ T_H_ cells had a moderate effect on tumor growth, but not on metastasis. This could be due to the fact that CD4^+^ T_H_ cells are instrumental to secrete cytokines that modulate CD8^+^ T_C_ cells and NK cells which in turn control tumor cells. Their role however is dispensable as other cells within the TME produce cytokines to modulate the professional killer cells [6, 7]. It has also been reported that low number CD8^+^ T_C_ cells are poor prognostic markers in non-small cell lung carcinoma and colorectal cancer development in humans [47] [48]. The number of metastatic foci in lungs of mice depleted of CD8^+^ T_C_ cells (Figure 7E) is less than a2V-KO mice (Figure 5C) might be due to the fact that other cell types are also affected (Figure 3A and Supplementary Figure 2C).

To our knowledge, this is the first report that demonstrates that the deletion of HSC-associated a2V has a severe effect on the distribution of αβ (CD4^+^ and CD8^+^) T cells in the peripheral blood and spleen (Figures 6A and 6B). In contrast, the distribution of other lymphoid cells like B cells and myeloid cells (data not shown) seems to be increased to maintain homeostasis. The exact mechanism for this defect in distribution of αβ T cells is currently unknown. Therefore, we analyzed the distribution of αβ T cells in the thymus and bone marrow and observed a defect in their development (Figures 6C and 6D). V-ATPase is a known pH sensor and it regulates pH of intracellular organelles and cytoplasm, controls endocytic traffic, regulates protein processing and degradation, and acts as a scaffold for protein-protein interactions [49]. Hence, any defect in a2V will also result in significant changes in the above mentioned mechanisms. Based on previous studies from our group, we are currently investigating various pathways to understand the reduced occurrence of αβ T cells in the bone marrow and thymus. Previously, it was shown that a2V deletion in the host mammary epithelial cells leads to defective glycosylation of extracellular matrix proteins due to the relocation of glycosyltransferase enzyme to endosomes [28]. We also have previously demonstrated that deletion of a2V in breast cancer and ovarian cancer cells leads to enhanced cell death by autophagy and apoptotic pathways, respectively, by increasing cytosolic pH [16, 50]. T cells originate in bone marrow, but develop and mature in thymus where they undergo positive and negative selection. During these selection processes a lot of T cells undergo apoptosis while relatively low number of mature T cells egress to circulation [51]. Furthermore, a defect in glycosylation in T cells block T cell progenitor renewal and clonal expansion [52]. Therefore, investigating cell death pathways and glycosylation pathways in the bone marrow and thymus might hold clues for solving this mystery.

**Figure 8:**
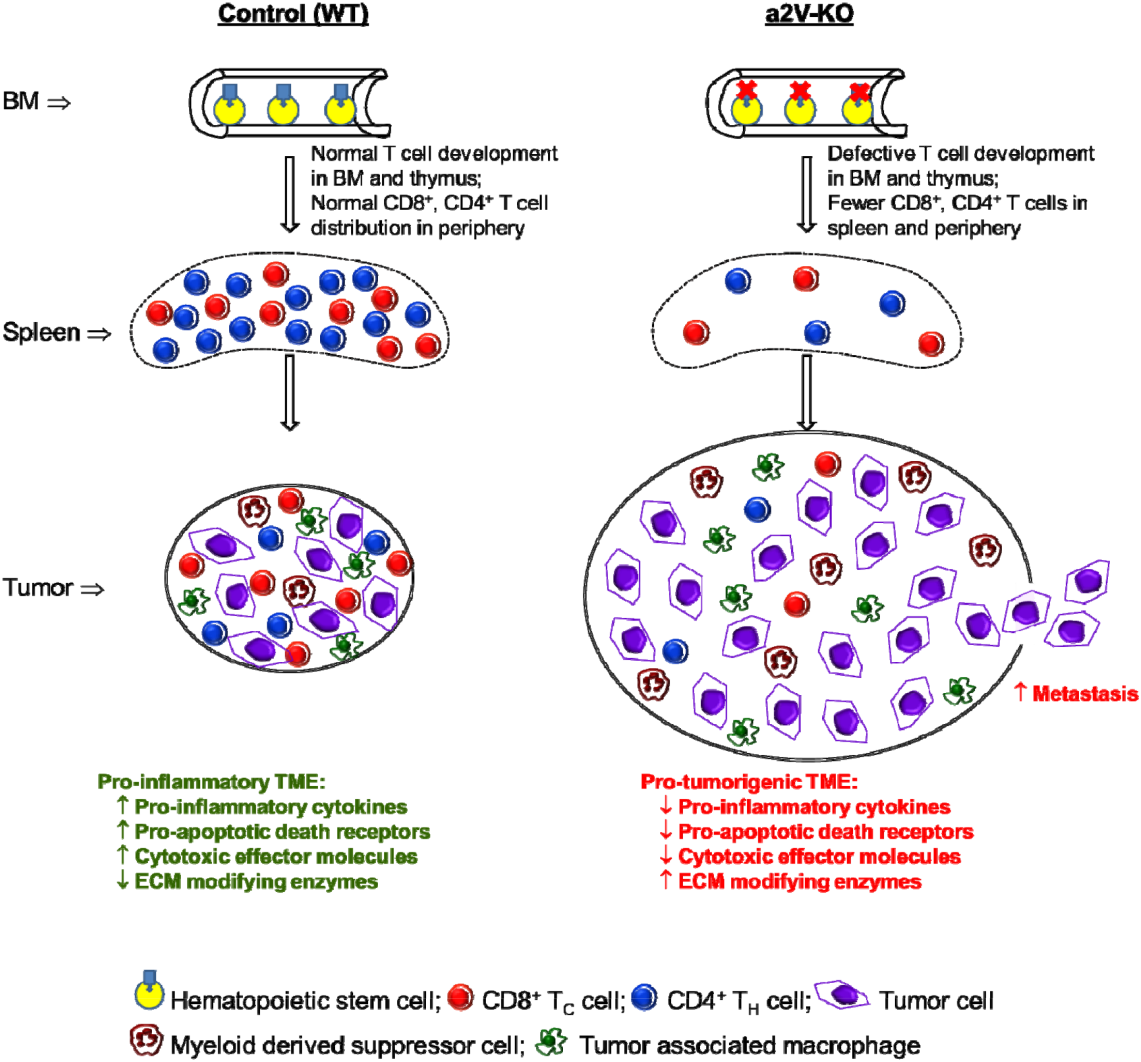
Proposed model and mode of action The deletion of a2V from the HSC results in a defect in αβ T production in the BM and thymus, which results in drastic reduction of αβ T cells in the periphery as represented in spleen. After tumor implantation, this reduction of αβ T cells promotes highly necrotic and metastatic breast tumors in the a2V-KO mice. The pro-tumorigenic TME of a2V-KO mice have lower levels of pro-inflammatory cytokines, death ligands, death receptors, cytotoxic effector molecules, CD4^+^ T_H_ cells, CD8^+^ T_C_ cells, higher levels of ECM modifying enzymes, and higher number of MDSCs compared to the pro-inflammatory TME of control mice. This pro-tumorigenic, immuno-suppressive microenvironment is conducive for faster tumor growth and allows tumor cells to metastasize.

In summary, this is the first time we demonstrate that the deletion of a2V from the HSC has a dramatic effect on the host immune system through reduction of CD4^+^ T_H_ cells and CD8^+^ T_C_ cells in lymphoid organs and periphery. This reduction of immune cell populations in the periphery and TME promoted necrotic breast tumors in the a2V-KO mice. The pro-tumorigenic TME of a2V-KO mice have significantly less pro-inflammatory cytokines, death ligands, death receptors, effector molecules, CD4^+^ T_H_ cells and CD8^+^ T_C_ cells compared to the control mice. Depletion of CD8^+^ T_C_ cells from the periphery of control mice also led to larger and metastatic tumors (Figure 8). Collectively, our findings show that V-ATPase activity in HSC is required for proper development of T cells and it can affect the progression of breast cancer. Further studies are required to decipher the mechanisms by which V-ATPase controls T cell development.

## MATERIALS AND METHODS

### Generation of a2V- knock out (a2V-KO) mice

All animal experiments were performed in the biological resource facility of Rosalind Franklin University of Medicine and Science (RFUMS) in accordance with the guidelines provided by the Institutional Animal Care and Use Committee (IACUC) of the National Institute of Health.

*ATP6v0a2* floxed (a2V^fl/fl^) mice were generated as described earlier [53]. Vav1^CreTg/0^ hemizygous mice were obtained from Jackson Laboratories (stock# 008610, Jackson Laboratories, Bar Harbor, ME, USA). To generate a2V-conditional knock out mice, a2V^fl/fl^ mice were crossed with Vav1^CreTg/0^ hemizygous mice. The resulting a2V^fl/0^Vav1^CreTg/0^ mice were further backcrossed with a2V^fl/fl^ (control) mice to generate a2V^fl/fl^Vav1^CreTg/0^ (a2V-KO) mice. Both control and a2V-KO mice are of the same C57BL/6 background. The presence of the a2V^fl/fl^ gene was confirmed by PCR using the following primer pair: forward primer 5’-AGGGTGGTGTCCTTTCACTCT-3’ and reverse primer 5’-ATCCCCAGGATCCACGCAT-3’. The presence of Vav1^Cre^ transgene was confirmed by the following primer pair: forward primer 5’-AGATGCCAGGACATCAGGAACCTG-3’ and reverse primer 5’- ATCAGCCACACCAGACACAGAGATC-3’ (IDT, IL, USA). The PCR conditions were followed as described in Jackson Laboratories website (Jackson Laboratories). The control and a2V-KO mice were followed up to 40 weeks of age and monitored for viability, fertility, growth or any externally visible abnormality.

### Tumor generation and antibody treatment

Breast tumors were generated as described earlier [19]. Briefly, 0.5x10^6^ of syngeneic E0771 (murine mammary adenocarcinoma) cells were suspended in 50μl HBSS and mixed with 50μl Corning Matrigel HC. This cell and Matrigel mixture was implanted into the 4^th^ mammary fat-pad of 8 week old control or a2V-KO female mice. For all experiments, littermate mice were used. The tumors were allowed to grow until day 14 post-injection and the length and width of the tumor was measured every alternate day. After 14 days post-implantation, the mice were euthanized, tumors were surgically removed, washed in PBS, weighed, and processed for further use. Tumor volume was measured by using the following formula: (LxWxWx0.5), where L= the longer measurement and W= the shorter measurement. Lungs were collected after perfusion with 10 ml of PBS and fixed in 16% formaldehyde solution (W/V).

To deplete total CD4^+^ T_H_ cells and CD8^+^ T_C_ cell populations, 8 wk old female control mice were injected (i.p.) with 250μg of anti-CD4 (clone-GK1.5), or anti-CD8 (clone-2.43), or isotype control (clone-LTF-2) from BioXcell (West Lebanon, NH). The mice received antibodies on the following days: D(-5), D(-1), D3, D7, D11 and D15. Tumor growth was measured until D21 post-implantation and mice were euthanized on that day, tumors were weighed and lungs were collected to examine for any metastasis by histology.

### RNA extraction and reverse transcription

RNA was isolated using Qiagen’s RNAeasy Mini Kit (Qiagen, CA). RNA was quantified using a Nanodrop (Thermo Fisher, CA). Reverse transcription was performed with 1μg of total RNA using Transcriptor First Strand cDNA Synthesis Kit (Roche, Switzerland).

### Quantitative reverse transcription PCR (qRT-PCR)

Fifty nanograms of RNA-equivalent cDNA was used for qRT-PCR using TaqMan Fast Advanced Master Mix (Applied Biosystems, Carlsbad, CA) and read with StepOnePlus real-time PCR instrument (Applied Biosystems). Validated TaqMan primer mix for a1V (Mm00444210_m1 Atp6v0a), a2V (Mm00441838_m1 Atp6v0a), a3V (Mm00469406_m1 Tcirg1) and a4V (Mm00459882_m1 Atp6v0a) were obtained from Applied Biosystems. The expression of test genes was normalized using internal house-keeping gene, GAPDH (4352339E-1208041).

### Targeted RNA-Seq via next-generation sequencing (NGS)

Next–generation sequencing (NGS) Library preparation was performed using Qiagen’s targeted “Mouse Inflammation and Immunity Transcriptome” panels containing probes for 485 genes as described earlier [54]. Briefly, cDNA was prepared from 400 ng of tumor RNA and unique molecular tags of 12 nucleotide length were incorporated into 20 ng cDNA via gene specific primer extension. After PCR purification using magnetic beads, the barcoded cDNA was amplified using gene specific primers. The purified DNA was again amplified through a second PCR reaction to insert index sequences that are unique to each sample. The completed library was loaded into Illumina’s reagent cartridge (150 cycle v3) and sequenced on a standard flow cell with custom sequencing primers provided by Qiagen.

Sequencing quality controls, including cluster density, total reads, and percent reads reaching Q30 were all within optimal ranges provided by Illumina. In addition, secondary quality controls provided by Qiagen’s targeted RNA-Seq software that reads and quantifies the sequencing files were all within acceptable ranges. The FASTQ files obtained from the sequencing runs were uploaded to Qiagen’s GeneRead DNAseq variant calling service. The data was then exported into a format that provides the total unique molecular barcode sequencing reads for each gene. All reads were normalized to 8 internal control housekeeping genes after screening negative for genomic DNA contamination. Mann-Whitney’s *U*-test was performed on the normalized data and expressed as “normalized expression” for the bar diagrams.

### Flow cytometry

Tumors were collected from tumor bearing mice on D14 after tumor inoculation and processed to get single cell suspension. Briefly, tumors were chopped to 1 mm size and incubated at 37°C with Collagenase IV (Worthington) and DNAse I (Sigma) for 2 hours while being rotated in RPMI medium without serum. The collagenase was neutralized with 10% fetal calf serum, washed twice with normal RPMI, filtered through 100μM cell sieve. The cell pellets from tumors were subjected to red blood cell lysing and filtered through a 70μM cell sieve. These cells were used to isolate the CD45^+^ cells using magnetically activated cell sorting (MACS, CD45 isolation kit, Miltenyi). Spleens, thymus, bone marrow and blood were collected from non-tumor bearing mice. Spleen, thymus and flushed bone marrow cells were mechanically disrupted to get a single cell suspension. These single cells and blood were subjected to red blood lysis and filtered through a 70μM cell sieve. The live, single cells were counted with trypan blue manually by a hemocytometer.

The single cells were subjected to live-dead staining (Live dead green kit, Applied Biosystems) and subsequently labeled with antibodies for flow cytometry for 30 min at 4°C. The following antibody cocktails were used: T cell cocktail containing antibodies against CD19, CD3, NKp46, TCRγδ, CD4, and CD8β; Myeloid cell cocktail containing antibodies for CD11b, GR1 or Ly6G, and F4/80. After washing in FACS rinse buffer, cells were fixed in 16% formaldehyde solution (W/V) for 20 min, washed again and resuspended in FACS rinse buffer (Biolegend). To identify different cell populations, cells were acquired by LSR II (BD) and analyzed by FlowJo software (Treestar, USA).

For analysis of lymphoid cells, live cells were gated on singlets (FSC-H vs FSC-A). The live cells further gated on expression of CD19 and CD3 and 3 gates are drawn. The CD19^+^CD3^-^ gate, CD19^-^CD3^-^ gate and CD19^-^CD3^+^ gate to represent B, non-B non-T and T cells, respectively. The non-B non-T gate was further analyzed for expression of NKp46 (FSC vs NKp46) and the CD19^-^CD3^-^NKp46^+^ cells were designated as NK cells. The T cells were further analyzed for γδ TCR expression. The CD19^-^CD3^+^γδ TCR^+^ and CD19^-^CD3^+^γδ TCR^-^ populations were designated as γδ T cells and αβ T cells, respectively. The CD19^-^CD3^+^γδ TCR^-^ αβ T cell populations were further analyzed for the surface expression of CD4 or CD8. The CD19^-^CD3^+^γδ TCR^-^CD4^+^ and CD19^-^CD3^+^γδ TCR^-^CD8^+^ cells were labeled as CD4^+^ T_H_ cells or CD8^+^ T_C_ cells, respectively. For analysis of myeloid cells, live cells were gated on singlets (FSC-H vs FSC-A). The live cells further gated for expression of CD11b. The CD11b^+^ cells were selected and further analyzed for expression of GR1 or Ly6G or F4/80 and were labeled as MDSC or TAN or macrophages, respectively.

### Histology and immunofluorescence analysis

Tumors and lungs were fixed in 16% paraformaldehyde solution (W/V) for 48 hour before embedding in paraffin. The paraffin embedded tissue blocks were sectioned 5μM thick, deparaffinized in xylene, stained with Mayer’s hematoxylin and 0.1% eosin, affixed coverslips with Permount mounting medium and visualized using a Leica ICC 50W light microscope (Leica Biosystems, Wetzlar, Germany).

For Immunofluorescence analysis (IFA) antigen retrieval was carried out on the deparaffinized sections by boiling in sodium citrate buffer (pH 6.0). The tissue sections were blocked to reduce background non-specific staining by blocking with 5% BSA and were incubated overnight with primary antibodies at 4°C. After washing with PBST, sections were incubated for 30 minutes with AF488-conjugated secondary antibodies at room temperature. Again after washing with PBST, sections were mounted using ProLong Diamond antifade mountant with DAPI (Applied Biosystems). After affixing coverslips, the slides were visualized with a Nikon EclipseTE2000-S fluorescence microscope (Nikon, Tokyo, Japan).

Freshly isolated hematopoietic stem cells (HSCs) were similarly processed for IFA in an Eppendorf tube, with exception to the antigen retrieval step. After staining with AF488-conjugated secondary antibody, the cells were spread on slides by cytospin, mounted as before with DAPI and visualized with a Nikon EclipseTE2000-S fluorescence microscope.

### Cell line and tissue samples

Murine mammary adenocarcinoma, E0771 cells (CH3 Biosystems, Amherst, NY, USA) which is syngeneic to C57BL/6 mice, were cultured as a monolayer in complete RPMI 1640 (Applied Biosystems) media supplemented with 10% fetal bovine serum, 100 U/ml penicillin, 100 U/ml streptomycin and 10 mM HEPES buffer at 37°C in a humidified 5% CO_2_ incubator.

Hematopoietic stem cells (HSCs) were isolated from mouse bone marrow. Both tibia and femur were collected from each leg of mice and the bone marrow was flushed out using DPBS. After lysing red blood cells, the HSCs were isolated using EasySep Mouse Hematopoietic Progenitor Cell Isolation Kit (Stem Cell Technologies, Canada) according to the manufacturer’s protocol. The purity of the pre- and post-sorted cells was examined by flow-cytometry using a cocktail of CD11b, CD3, CD19, GR1, B220, and Ter119 antibodies (Biolegend).

### Statistical analysis

All data are represented as mean ± SEM unless stated otherwise. Mann-Whitney *U* test or unpaired *t*-test was used to calculate the statistical significance between groups as specified in the figure legends. Significance was set at *p*<0.05. Analysis was performed using GraphPad Prism 5 software (GraphPad Prism Software Inc., La Jolla, CA).

## SUPPLEMENTARY MATERIALS FIGURES



## Abbreviations

a2V: a2 isoform of vacuolar ATPase
HSC: Hematopoietic Stem Cell
KO: Knock out
MDSC: Myeloid derived suppressor cell
TAM: Tumor associated macrophage
TAN: Tumor associated neutrophil
T_H_ cell: T helper cell
T_C_ cell: Cytotoxic T cell
TME: Tumor micro environment
