# Supplementary material for: Hematopoietic stem cell specific V-ATPase controls breast cancer progression and metastasis via cytotoxic T cells
Source: Oncotarget. 2018 Sep 4;9(69):33215–31. doi: 10.18632/oncotarget.26061 (PMC6145706; doi:10.18632/oncotarget.26061)
Supplement: Supplementary file 1 [file oncotarget-09-33215-s001.pdf]

Hematopoietic stem cell specific V-ATPase controls breast cancer progression and metastasis via cytotoxic T cells

SUPPLEMENTARY MATERIALS

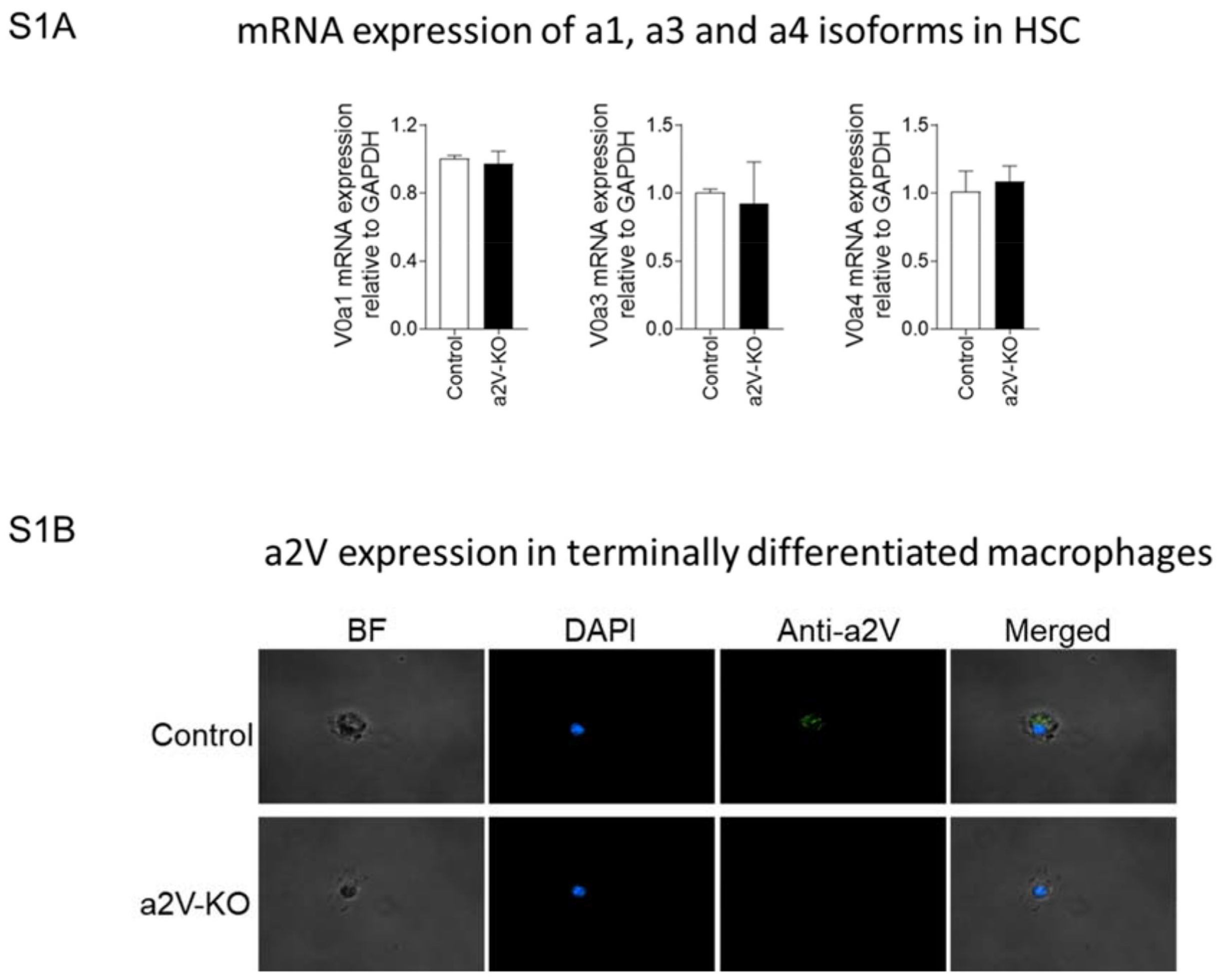

Supplementary Figure 1: Hematopoietic cells lack a2V expression in a2V-KO mice. **(A)** Relative mRNA levels of V0a1, V0a3, and V0a4 isoforms of V-ATPase in HSCs are shown. Mouse GAPDH is used as an endogenous control for normalization. Data is represented as mean ± SEM (n=6, Mann-Whitney *U* test). **(B)** Representative IFA image showing lack of a2V protein (green fluorescence) in bone marrow derived macrophage (an example of terminally differentiated cell of hematopoietic origin), from a2V-KO mice (n=3, 40X magnification).

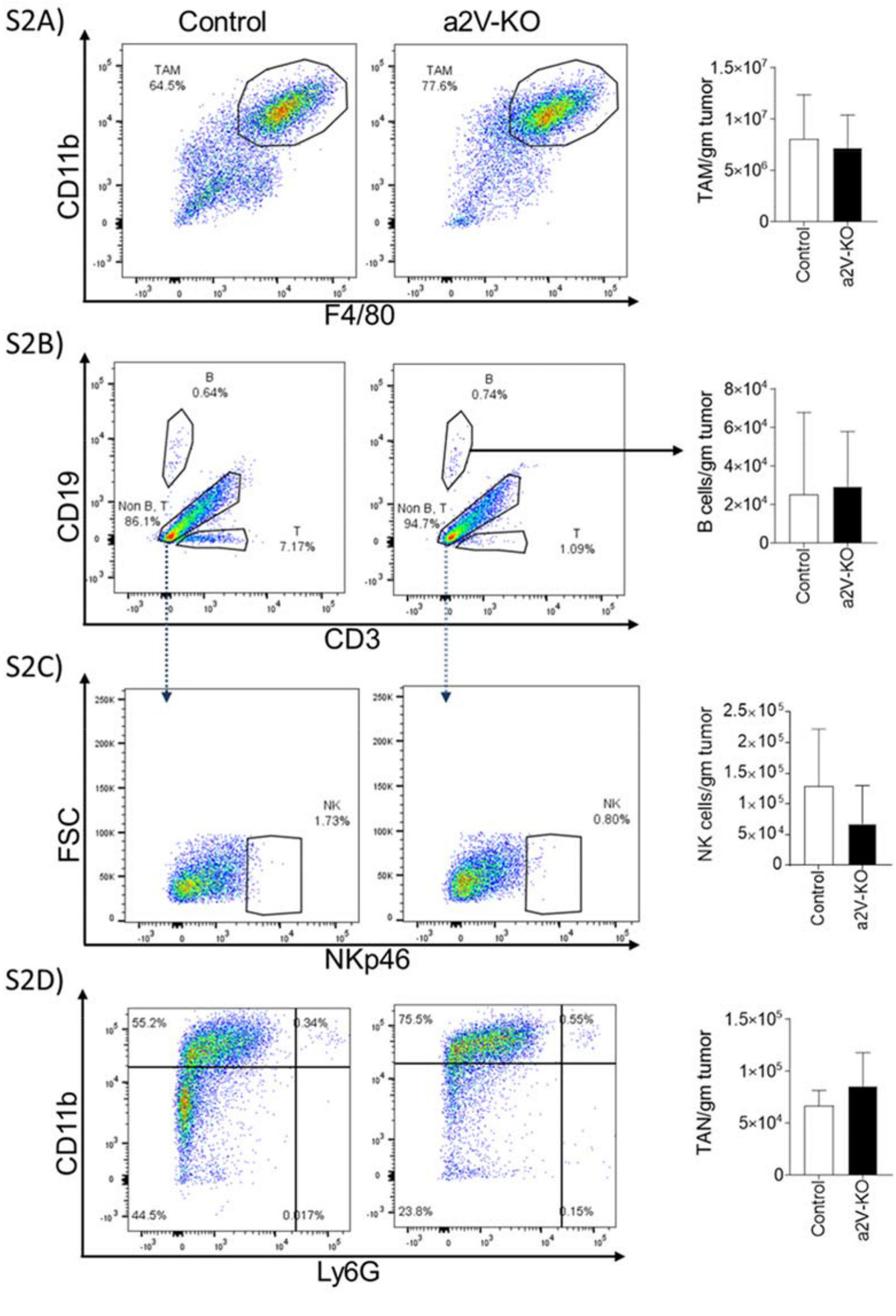

Supplementary Figure 2: a2V deletion in HSCs cause altered recruitment of immune cell populations into the TME. The single cells from tumors were enriched for CD45 expression by MACS and subjected to flow-cytometry. Representative histograms are shown in the left side. Bar graphs show the number of single, live, $CD45^+$ cells per gram of tumor. **(A)** Tumor associated macrophages (TAMs) **(B)** B cells **(C)** NK cells Pooled results from three independent experiments with control n=14 and a2V-KO n=11, mean ± SEM, Mann-Whitney *U* test. **(D)** Tumor associated neutrophils (TANs), control n=5 and a2V-KO n=5, mean ± SD, Mann-Whitney *U* test.

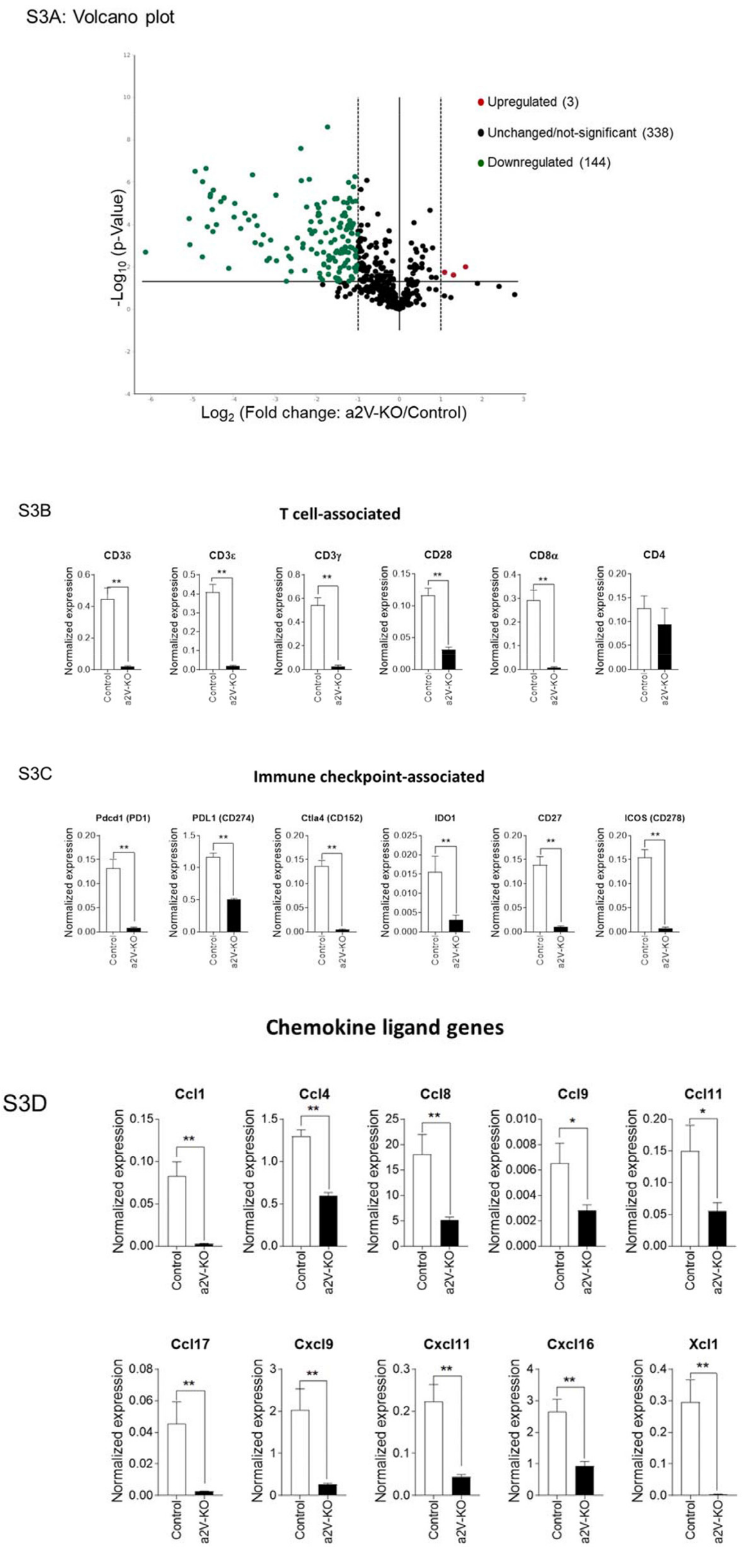

Supplementary Figure 3: Analysis of the TME by targeted RNASeq. Equal amounts of tumor RNA was used for targeted RNASeq and data were analyzed using Qiagen's data analysis center. Eight housekeeping genes were used to normalize gene expression. **(A)** Volcano plot of all the analyzed genes. The X-axis represents log base 2 of fold change of transcripts in a2V-KO TME compared to control TME. The Y-axis represents negative log base 10 of the *p*-value of the analysis. The transcripts with more than 2 fold up- or down-regulation are shown to the right or left of the vertical dotted lines, respectively. Data with $p \leq 0.05$ was considered significant. Bar graphs show **(B)** T cell-associated genes **(C)** Immune checkpoint-associated genes **(D)** Chemokine ligand genes. Pooled results from two independent experiments with n=6, mean ± SEM, Mann-Whitney *U* test, $^{*}p<0.05$, and $^{**}p<0.01$).

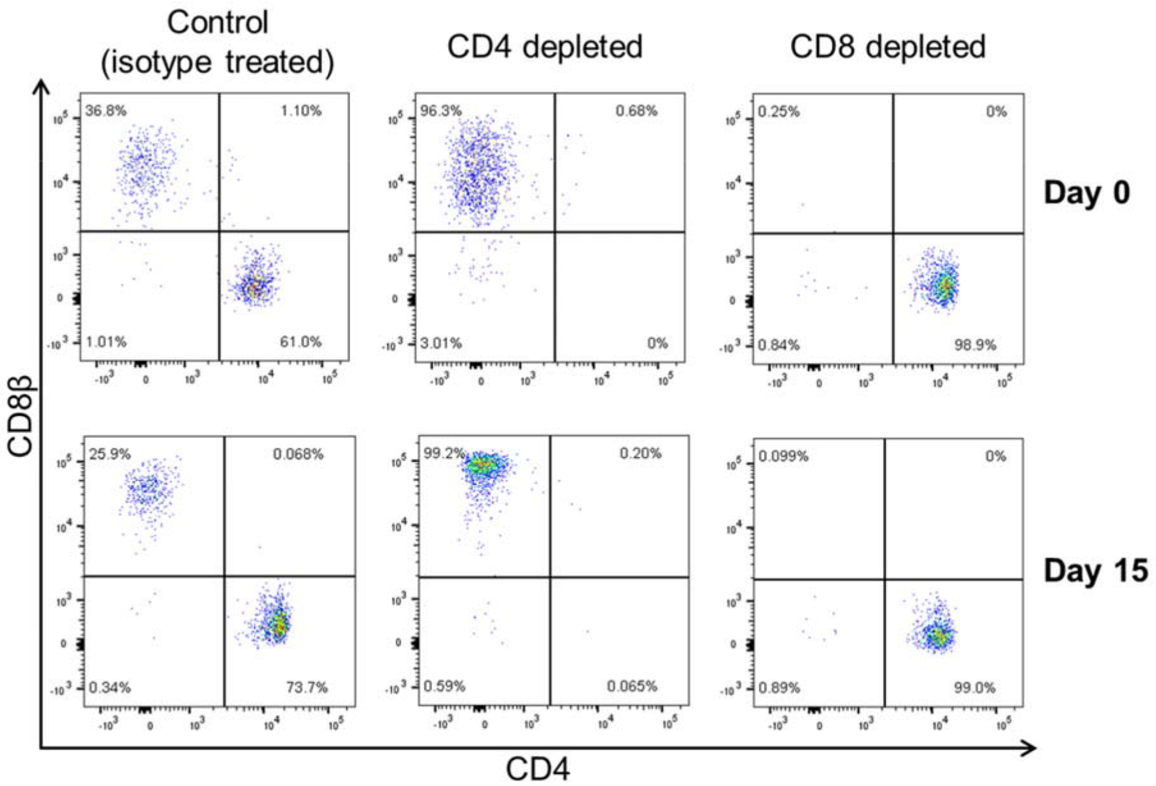

Supplementary Figure 4: Status of CD8 and CD4 cells in blood on the day of tumor implantation (D0) and on Day 15. Representative histograms of live $CD19^-CD3^+\gamma\delta$ TCR^- cells showing distribution of $CD4^+$ T_H cells and $CD8^+$ T_C cells in peripheral blood of mice on day of tumor implantation (Day 0, upper panels) and day 15 post-transplantation (lower panels). Blood was collected with EDTA to prevent clotting. After RBC lysis, live-dead staining, and surface staining with antibodies, the cells were analyzed by flow cytometry.
